# Structural Studies of the Phage G Tail Demonstrate an Atypical Tail Contraction

**DOI:** 10.3390/v13102094

**Published:** 2021-10-18

**Authors:** Brenda González, Daoyi Li, Kunpeng Li, Elena T. Wright, Stephen C. Hardies, Julie A. Thomas, Philip Serwer, Wen Jiang

**Affiliations:** 1 Department of Biological Sciences, Hockmeyer Hall of Structural Biology, Purdue University, 240 South Martin Jischke Drive, West Lafayette, IN 47907-1971, USA; gonzalb@purdue.edu (B.G.); li3221@purdue.edu (D.L.); li2182@purdue.edu (K.L.); 2 Department of Biochemistry and Structural Biology, The University of Texas Health Center at San Antonio, 7703 Floyd Curl Drive, San Antonio, TX 78229-3900, USA; wrighte@uthscsa.edu (E.T.W.); hardies@uthscsa.edu (S.C.H.); serwer@uthscsa.edu (P.S.); 3 Gosnell School of Life Science, Rochester Institute of Technology, 85 Lomb Memorial Drive, Rochester, NY 14623, USA; jatsbi@rit.edu

**Keywords:** myophage, tail contraction, tail sheath, anchor

## Abstract

Phage G is recognized as having a remarkably large genome and capsid size among isolated, propagated phages. Negative stain electron microscopy of the host–phage G interaction reveals tail sheaths that are contracted towards the distal tip and decoupled from the head–neck region. This is different from the typical myophage tail contraction, where the sheath contracts upward, while being linked to the head–neck region. Our cryo-EM structures of the non-contracted and contracted tail sheath show that: (1) The protein fold of the sheath protein is very similar to its counterpart in smaller, contractile phages such as T4 and phi812; (2) Phage G’s sheath structure in the non-contracted and contracted states are similar to phage T4’s sheath structure. Similarity to other myophages is confirmed by a comparison-based study of the tail sheath’s helical symmetry, the sheath protein’s evolutionary timetree, and the organization of genes involved in tail morphogenesis. Atypical phase G tail contraction could be due to a missing anchor point at the upper end of the tail sheath that allows the decoupling of the sheath from the head–neck region. Explaining the atypical tail contraction requires further investigation of the phage G sheath anchor points.

## 1. Introduction

Phage G’s virion is remarkable because of its size—it is recognized as the largest phage that has been laboratory-propagated [1,2,3]—which raises many questions as to how such a large structure assembles and maintains stability. In our recent report on phage G, we analyzed the DNA-containing head using cryo-electron microscopy (cryo-EM). From these data, we observed heterogeneity in the dsDNA density in the head, and we obtained the structure of its T = 52 icosahedral capsid to a 6.1 Å resolution, which highlighted the HK97-like major capsid protein and the lambda-like decoration trimers [4]. Additionally, there was heterogeneity in the dsDNA density [4] of its 498 kbp genome [2,5,6]. 

In the current study, we focused on the phage G tail structure. Our interest in analyzing phage G’s tail stemmed from our initial negative stain EM analysis of phage G–host images, in which the tail sheath appeared decoupled, post-contraction, from the head–neck region; this was contrary to the results previously reported for the well-studied phage T4 and other myophages [5,6,7,8,9,10,11]. In myophages, the typical contraction of the tail sheath happens upwards, toward the neck, and the tail tube protrudes through the bottom of the baseplate into the host cell (or becomes exposed if the particle is not adsorbed to a host cell) [5,6,7,8,9,10,11]. Phage G’s tail had previously been studied by negative stain EM over five decades ago by the researchers that discovered and isolated phage G, but information since then about phage G’s tail has been limited [3,12,13,14,15].

The early studies of phage G by Donelli et al. in 1972 found that the tail was approximately 450 nm in length, and was composed of a 6-start, right-handed helix [15]. Based on this analysis, it was hypothesized that both the non-contracted and contracted tail sheaths had helical symmetry. The original structural studies from the 1970s also reported that, upon contraction, the tail sheath underwent a compression of 22.6 Å in rise along the axial direction, with a change in twist around the axis by 6.6° per subunit [15]. However, likely due to the challenges that come with helical reconstruction from electron micrographs [16], cryo-EM 3D reconstruction analysis has not yet been applied to the phage G tail. Here, we perform this analysis after presenting observations on the unusual contraction of the phage G tail. We also present studies to probe the evolutionary relationship of phage G relative to other tailed phages. 

## 2. Materials and Methods

### 2.1. Phage G Propagation and Purification

Phage G and its host were obtained from the Fangman lab (University of Washington, Seattle) [17], which had acquired them from the Donelli lab (Istituto di Fisica, Rome) which first isolated phage G [3]. Phage G was grown and isolated as previously described [4,18]. Briefly, phage G was amplified as a plate stock in agarose overlays, which were collected, and large debris was cleared by centrifugation. Phage G was then purified by rate zonal centrifugation in a sucrose gradient and stored in 0.01 M Tris–Cl (pH 7.4), 0.01 M MgSO_4_, and 6% polyethylene glycol MW 3350.

### 2.2. Negative Stain EM of Phage G–Host Interaction

We used negative stain EM to observe phage G attached to its host strain (PGH) bacterium, recently revised to *Lysinibacillus* sp. [4]. The cells were grown to mid-log phase in TB media (10 g tryptone, 5 g NaCl in 1000 mL H_2_O) [19] and were infected at an MOI of 1 and monitored by OD600 nm until lysis occurred. The lysed and remaining cells were then spun down at 5000× *g* for 10 min and washed twice in PBS buffer (pH 7.0) and resuspended to a final OD600 = 1. The resuspended content was then deposited onto lacey carbon grids coated with graphene oxide and stained with sodium phosphotungstate (PTA) solution for negative stain EM. The grid was imaged on a Tecnai T20 200 kV Electron Microscope (FEI, Hillsboro, OR, USA) using a US1000 2Kx2K CCD camera (Gatan, Pleasanton, CA, USA).

### 2.3. Cryo-EM Data Collection

The cryo-EM data collection procedure for this study was previously described in Gonzalez et al., 2020 [4]. Briefly, a 3 μL drop of purified phage G was put onto a 400 mesh Ted Pella ultrathin lacey carbon grid and incubated for 30 min in a humid chamber on ice. The grid was then washed with 10 μL of buffer (0.01 M Tris–Cl (pH 7.4), 0.01 M MgSO_4_, and 6% polyethylene glycol MW 3350). The grid was frozen using a Gatan CP3 plunger, where it was initially blotted for 9 s with Whatman #1 filter paper at 65% humidity, and then plunge-frozen in liquid ethane.

The frozen phage G grid was then imaged using the Titan Krios (FEI, Hillsboro, OR, USA) equipped with a Gatan K2 Summit direct electron detector (Gatan, Pleasanton, CA, USA) in super-resolution mode at Purdue Cryo-EM Facility with 8700× nominal magnification, and a sampling of 1.742 Å/super-resolution pixel. Overall, 375 movies were collected, which were then motion corrected using Motioncorr [20]. The movie averages were 2× binned to 3.484 Å/pixel for further image processing. Detailed data collection parameters are listed in Supplemental Table S1.

### 2.4. Categorization of Tail Contraction States from Cryo-EM Micrographs

In our cryo-EM dataset, it is apparent there are multiple phage G tail states even in the absence of the host. To further explore these states, we visually classified particles with complete tails as either contracted or non-contracted. Furthermore, we observed whether the contracted tail sheath was present either near the head, in the middle section of the tail, or near the tail tip. 

### 2.5. Helical Reconstruction of Non-Contracted and Contracted Phage G Tail Sheath

Phage G tail was manually picked using the Relion helical picker [21]. Helical segments were extracted with an 18.7 Å rise and 5 asymmetric subunits using a 224-pixel box. In total, 22,755 segments were extracted (Table S2). To obtain the initial helical twist and rise values of phage G tail components, the extracted segments were used in CryoSPARC for further analyses [22]. The tail tube, the non-contracted, and the contracted tail sheaths were visually separated based on 2D classification results and used for ab initio modeling, specifying C6 symmetry. From there, a relatively low-resolution 3D model was generated that could then be used to analyze the helical symmetry (e.g., twist and rise) parameters using the helicalSym.py program from the jspr package [23]. 

To further improve the 3D reconstructions, several additional rounds of 2D classification were conducted to remove heterogeneity. Helical refinement was then conducted using CryoSPARC to generate refined, helical models for the non-contracted and contracted tail sheath structures. All parameters used for the helical refinement were default in the contracted phage G helical reconstruction, except for the following: 27.13° twist, 18.89 Å rise, 15 maximum symmetry order, and C6 symmetry. All parameters used for the helical refinement of the non-contracted phage G helical reconstruction were default, except for the following: 20.57° twist, 41.53 Å rise, 40 maximum symmetry order, and C6 symmetry. The 3D maps of the non-contracted and contracted tail sheaths are deposited to the Electron Microscopy Data Bank (EMDB) with accession IDs, EMD-25155 and EMD-25154, respectively.

### 2.6. Bioinformatic Evolutionary Analysis of Phage G Tail Sheath

A protein sequence-based evolutionary analysis of the phage G sheath protein, gp178, was performed as described previously [24]. Briefly, the T02 aligner [25,26], obtained from the U.C.S.C bioinformatics group (https://compbio.soe.ucsc.edu/sam.html, version 3.5 obtained 1 December 2017), was used to align a homologs set, which was generated from the union of PsiBlast hits keyed with diverse known myoviral sheath protein sequences. A subset of sheath proteins covering a broad representation of the resulting tree was then selected for refinement. The quality of alignment across two of the most divergent lineages (T4 to Bxz1, and Bxz1 to phage G) was checked by HMM–HMM comparison using HHpred [27]; this resulted in limiting the final tree to the region corresponding to residues 357–655 of T4 gp18, and in the removal of sequences with large numbers of gap characters. A further check, to avoid perturbation of the tree by recombinant or partially aligned sequences, was to divide that region in two and remove any sequences not producing a congruent tree in the two subsections. The final tree was calculated using MrBayes [28] with an independent gamma rate relaxed clock model [29]. The time scale was set by the alignment of nodes in the SPO1–LP65–Bastille clade and the T4–Aeh1–KVP40–Syn1 clade, with a scaled large terminase tree as described [24].

## 3. Results

### 3.1. Negative Stain EM of Phage G Host Attachment

The goal of these analyses was to gain a better understanding of the phage G tail structure and its role in infection. Our interest in phage G’s tail sheath was initially piqued by a preliminary TEM of negatively stained phage G particles adsorbed to the host cell wall. In those micrographs, we observed numbers of particles whose tail sheaths were contracted in a manner that was not consistent with previous descriptions of the tail behavior of other myoviruses, where the contracted tail sheath is always coupled to the neck region [5,6,7,8,9,10,11]. Those initial results led us to perform a cryo-EM reconstruction of the phage G tail sheath to gain a better understanding of the structural basis for this behavior.

**Figure 1:**
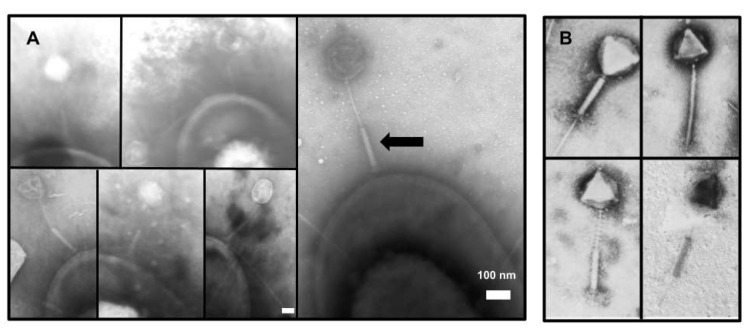
TEM of negatively stained phage G showing multiple states of the contractile sheath. (**A**) Phage G particles attached to the host cell and (**B**) Micrographs of phage G virions, reproduced from Ageno et al., 1973 [12]. In (**A**), all host cell-adsorbed phage G particles had the tail sheath contracted toward the head–distal tip, which was in contact with the host cell surface. The arrow indicates an example of one sheath contracted in this atypical manner. The white bar represents 100 nm in length. In (**B**), images from [12] show that the phage G tail sheath contracted at different positions along the tail (next to the head, at the middle of the tail, and near the tail tip), demonstrating the atypical behavior of the phage G sheath that we observed both by negative stain TEM and cryo-EM (see below). Images in (**B**) used with permission from Elsevier, Copyright (1973).

The TEM of a negatively stained phage G and its host revealed particles adsorbed to the host surface (Figure 1A). Sometimes, the head did not have strong DNA density—suggesting genome ejection to initiate an infection. All the phage G particles observed appeared to have a contracted tail conformation with the tail sheath, located at the head–distal end of the tail, in contact with the host surface via the tail fibers (Figure 1). This is in stark contrast with typical observations of contracted myophages, such as T4, where the contracted tail sheaths are located at the opposite end, next to the head [5,6,7,8,9,10,11]. To find out if these unusual observations of the contracted tail sheath near the tail tip are unique to our data, we examined the literature. We found multiple negative stain images of purified phage G particles in which the contracted phage G tail sheath was seen at different positions along the tail (attached to the head, at the middle of the tail, and near the tail tip) (Figure 1B). The localization of the contracted tail sheath near the tail tip was not discussed by the authors [12]. 

### 3.2. Tail Contraction States in Our Cryo-EM Data

To test the possibility that negative staining caused these atypical results, we collected single particle cryo-EM images of purified phage G particles. We observed multiple states of tail contraction among the phage G particles, in agreement with the earlier observations noted above (Figure 1B). We manually categorized phage G particles from our cryo-EM derived micrographs based on the morphology of the tail sheath and its location along the tail. All phage particles with completely visible, easily distinguishable tails were counted, resulting in 364 distinct phage G particles analyzed. 

**Figure 2:**
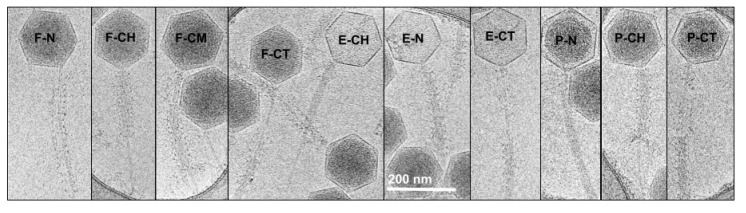
Representative members of cryo-EM-based categories of purified phage G. Particles are labeled as follows: F—DNA full; P—partial DNA; E—empty; N—non-contracted; CH—contracted near head; CM—contracted middle; CT—contracted at tip, as shown by the representative images.

The tails were then categorized based on: (i) The tail contraction state; (ii) The location of the contracted tail sheath relative to the head; (iii) The head state; these are summarized in Table 1. Examples of each state described in Table 1 are shown in Figure 2. The two contraction states are non-contracted (uniform thickness along the entire tail) and contracted (variable thickness along the tail). The locations of contracted tail sheath are grouped into 3 places: near-head, middle, and near-tip. Separately, apparent DNA-full or DNA-empty head categorizations were assigned visually.

Overall, there were 211 particles (58%) that were identified as non-contracted, with the remaining 153 (42%) phage G particles having contracted tail sheath states. The tail sheath contractile states did not appear to be significantly influenced by the presence or absence of DNA in the head. Among the DNA-full phages, 40% (132 of 328) of tails exhibited contraction, whereas among the partial DNA and DNA-empty phages, 58% (21 of 36) exhibited contraction (Table 1). 

The most unusual observation was that 80% (123 of 153) of the contracted-sheath particles had the sheath detached from the neck region and in contact with the baseplate at the distal tip of the tail. Only 18% of the contracted particles had the tail sheath located under the neck region. The remaining 2% had the tail sheath in the middle of the tail. The contracted states, with the tail sheath near the middle of the tail or at the distal tip of the tail, were not observed for other myophages, such as T4, in non-natural conditions [30].

### 3.3. Phage G Tail Components from 2D Classification

**Figure 3:**
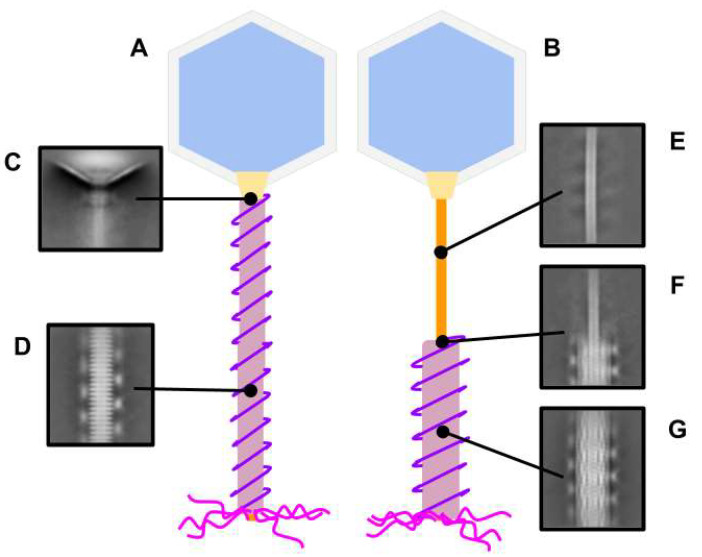
2D classification of phage G tail components. The cartoons above represent the non-contracted (**A**) and contracted (**B**) tail sheath states of phage G. The small insets are 2D classifications from the cryo-EM data that point to the corresponding tail components in the cartoons as follows: (**C**) neck region; (**D**) non-contracted sheath; (**E**) tube; (**F**) contracted sheath and tube junction; (**G**) contracted sheath.

The various tail contraction states of phage G raised questions about the structure and changes of the tail sheath subunits in phage G; thus, we obtained tail structures using single-particle cryo-EM analysis. Tails were selected from micrographs using the manual helical particle picking tool in Relion [21] from the head–tail junction down past the tail tip. From there, segments were extracted using the Relion helical segmenting tool [21], with a 93.5 Å interbox distance and a 224 pixel box size. In total, 22,755 segments were extracted. Using CryoSPARC [22], the particles were then subjected to multiple rounds of reference-free 2D classification (details in the Table S2). From the 2D classification results, we were able to detect different phage G tail components, including: the neck region (Figure 3C), the non-contracted tail sheath (Figure 3D), the tail tube (Figure 3E), the tail tube and contracted tail sheath junction (Figure 3F), and the contracted tail sheath (Figure 3G). 

**Figure 4:**
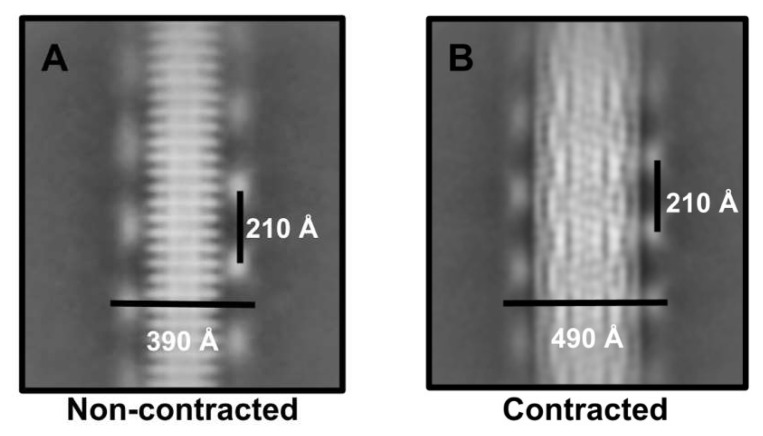
Phage G outer coil density in 2D classification. Density from the outer coil was observed in phage G sheaths that were (**A**) non-contracted, and (**B**) contracted. In both sheath states, the outer coil had the same measured axial pitch distance (210 Å).

During the 2D classification analysis, we observed density on the side of the tail sheath that corresponds to the outer coil density that was described in Donelli et al.’s 1972 negative stain EM study [15]. This is a unique feature that, to our knowledge, has not been observed in other phages. To take a closer look at this feature, we measured the distances between the peaks of density and assigned it to the outer coil diameter and helical pitch (Figure 4). 

In the non-contracted tail sheath, the measured outer coil diameter was 390 Å. In the contracted tail sheath, the outer coil diameter increased to 490 Å (Figure 4). The axial distance between the peaks of outer coil density were the same (210 Å) in both tail sheath states (Figure 4). In other words, the outer coil maintains its axial pitch while its diameter undergoes significant changes during contraction. 

This suggests that neither end of the outer coil is coupled to the tail sheath. Instead, the upper end of the outer coil is coupled to the neck region and the bottom end is coupled to the baseplate/tail tube—to maintain the total length and helical pitch during sheath contraction—while its diameter was forced to widen by the contracted sheath. The outer coil density was not well resolved in our cryo-EM reconstructions of the tail sheath and had a blurred appearance in the 2D classification results. We speculate that these results are a consequence of the coil having different helical symmetry to the tail sheath (Figure 4).

### 3.4. 3D Cryo-EM Structure of the Non-Contracted and Contracted Phage G Tail Sheath 

**Figure 5:**
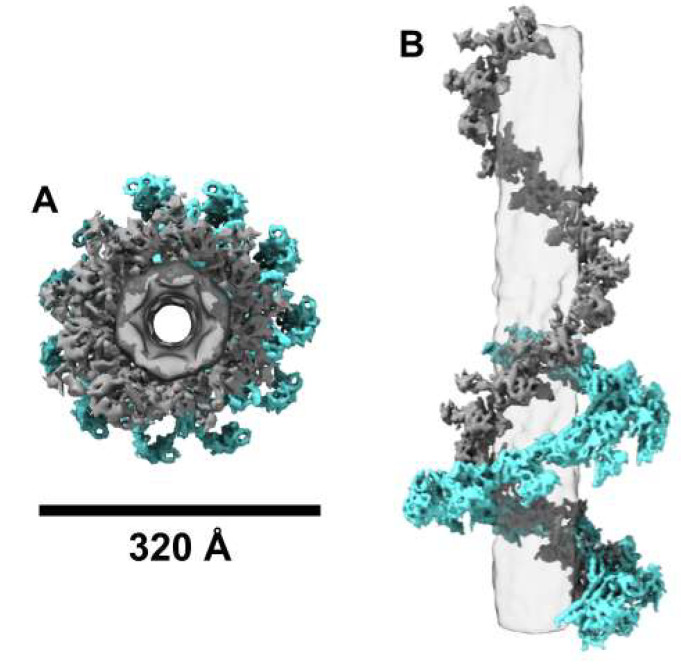
Non-contracted and contracted organization of the phage G tail sheath. Representation of 16 tail sheath subunits from a single strand of the six-stranded tail sheath of phage G in the non-contracted conformation (grey), and in the contracted conformation (cyan) cryo-EM structures in the top (**A**) and side (**B**) views. The tail tube density is shown in transparent grey.

The 2D classification results of phage G highlighted the heterogeneity and variety in its tail states. The segments from 2D classes identified as non-contracted and contracted tail sheaths were subjected to 3D helical reconstruction using CryoSPARC [22]. The resolution of both the non-contracted and contracted tail sheath cryo-EM density was approximately 7–8 Å, and 6–7 Å, respectively (Figure S1). The outer and inner diameter of the non-contracted tail sheath of phage G is approximately 240 Å and 60 Å, respectively (Figure 5 and Table 2). After contraction, the outer and inner diameter of phage G’s tail sheath expands to 320 Å and 120 Å, respectively (Figure 5 and Table 2). The tail sheath proteins of phage G are arranged in a six-strand, right-handed helix around the inner tail tube structure (shown in transparent grey in Figure 5), through which the dsDNA is transferred during infection. The tail sheath proteins in the non-contracted state, in each helical strand, are organized with a symmetry of 20.57° twist and 41.53 Å rise (Figure 5 and Table 2). 

The contracted tail sheath structure maintains the six-stranded, right-handed helical arrangement, but the sheath proteins compact to form a helix with helical symmetry of 27.13° twist and 18.89 Å rise (Figure 5 and Table 2). After the tail sheath contraction, the outer diameter of the sheath widens by 80 Å and the inner diameter doubles. The total length of the phage G tail is 4500 Å, and the non-contracted tail sheath organization has a rise of 41.53 Å; therefore, ~648 sheath subunits are arranged on the tail in total. From this, the calculated length of the completely contracted phage G tail sheath would be 108 subunits per strand, multiplied by an 18.89 Å rise per subunit, which is 2040 Å. This is consistent with our observations of the phage G contracted state from our negative stain and cryo-EM micrographs, where about half of the tail length is occupied by the contracted tail sheath (Figure 1 and Figure 2). 

### 3.5. Phage G Tail Sheath Subunit Structure and Arrangement

**Figure 6:**
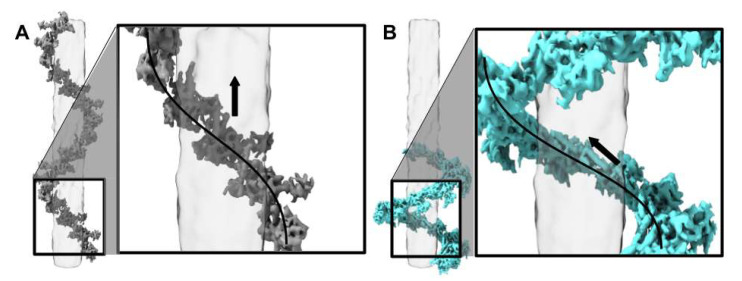
Tail contraction twists the orientation of individual phage G tail sheath subunits with no impact on intra-subunit structure: (**A**) non-contracted; (**B**) contracted. There are contacts with two long alpha helices in the inner density of the tail sheath (grey) in parallel with the tube (transparent grey). The arrow indicates the direction of the two alpha helices. In the contracted state, sheath proteins twist approximately 30°. In both (**A**) and (**B**) the phage head would be at the top of the figure.

**Figure 7:**
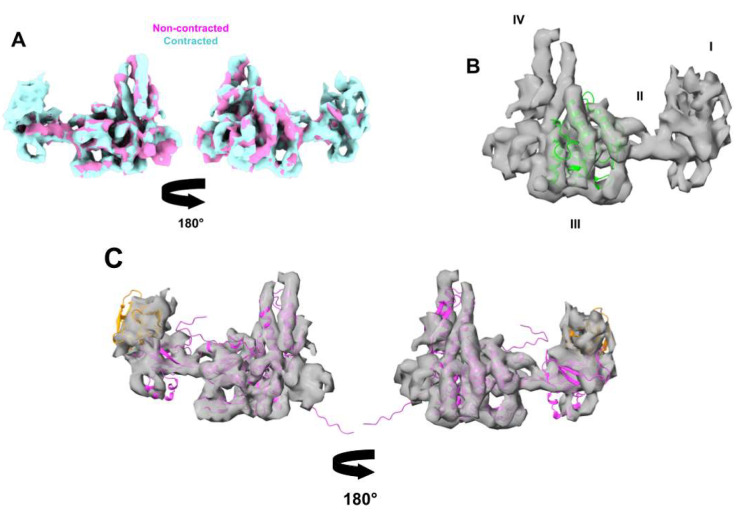
Phage G tail sheath subunit structure. (**A**) The phage G tail sheath subunit from the non-contracted (pink) and contracted (cyan) structures were superimposed using ChimeraX fit-to-model feature [34]. The densities superimposed almost completely, except for the outer domain region, where the non-contracted structure (pink) has poorly defined density. (**B**) The phage G sheath protein structure has several alpha helices as shown by the rodlike density of the contracted tail sheath subunit (transparent grey). The phage G sheath structure also follows the same domain organization described for T4 [6,33]. The green alpha helices were fitted from the T4 tail sheath crystal structure residues 21–201 (PDB: 3FOA) [6]. (**C**) Alphaflold2 [32] was used to generate a model prediction of the phage G sheath protein and the two regions (magenta and orange) were fitted independently into the contracted phage G sheath subunit density. The magenta area (residues 1–119 and 219–579) represents the innermost domain regions, and the outermost domain is in orange (residues 120–218). The first 26 and last 17 amino acids were predicted to be disordered.

Phage G’s tail sheath-forming protein (gp178) is 579 amino acids long and has a predicted mass of 63 kDa based on its protein sequence (AEO93438.1). The sheath proteins form a six-stranded, right-handed helix that wraps around the tail tube structure. The phage G sheath protein has a relatively large core domain close to the tail tube, and a smaller, outwardly protruding domain (Figure 6 and Figure 7). Within the core domain, multiple rodlike densities of alpha helices are resolved (Figure 7). There are two main alpha helices that are closest in proximity to the tube (the tube is transparent grey Figure 6). The outwardly protruding domain is less well-resolved than the inner core region (Figure 7A) and appears to have less alpha helical density.

At the domain level, phage G’s sheath protein appears to be similar to the sheath protein of T4, gp18 [6], as labeled in Figure 7B. From the partial crystal structure of T4, the four domains I, II, III, and IV are organized from most exposed to most buried towards the tube, respectively [6]. Domain I (residues 98–188) has a six-stranded beta-barrel and an alpha helix [6]. Domain II is then composed of a two-layer beta sandwich, surrounded by four alpha helices, and is defined by residues 88–97 and 189–345 [6]. Domain III is then described as a beta sheet with six beta strands and six alpha helices and is defined by residues 20–87 and 346–510 [6]. Finally, domain IV has not been resolved in the T4 crystal structure, but is composed of the termini (residues 1–20 and 510–659) [6]. Cryo-EM structural studies on phi812 also found that its sheath protein, gp103, had a similar organization at the individual sheath protein subunit level to T4 (Figure S2) [11]. In phi812, the cryo-EM density in the domain IV region has been described to contain 2 major helices [11]. The region has not been described from the T4 studies because of the limited structural information [8,31,32], but we have also observed these two helices in our phage G gp178 cryo-EM density (Figure 6 and Figure 7).

As in the sheath protein gp18 of phage T4, in phage G’s gp178 sheath structure, domain I is the outermost facing region of the sheath (Figure 7B). It is also the least resolved portion of the sheath protein density (Figure 7A). The density for domain II is not as large as it is described for T4 [31,33], which could be due to phage G’s sheath protein, gp178 (579 amino acids), being smaller than T4’s gp18 (659 amino acids) [31]. In phage G, as for T4’s gp18, domain III is mainly alpha helical (Figure 7B) [6]. In domain III of phage G’s density, a prominent bundle of three alpha helices matches well with T4’s helices in the same region (Figure 7B). 

We used Alphafold2 [32] to predict the structure of phage G’s sheath protein (gp178) (Figure 7C). In the predicted structure, the orientation of the outermost domain, relative to the main core region, did not match the segmented density from our phage G contracted sheath structure. For this reason, we have fitted both domains independently into the density (Figure 7C). The outermost domain from the predicted structure (residues 120–218) is shown in orange in Figure 7C, and the rest of the predicted structures are in magenta (residues 1–119 and 219–579) in Figure 7C. The predicted core domain fits the EM densities well, including the matching of the helices (Figure 7C). The first 26 and last 17 residues of the structure did not have a predicted fold, and therefore are shown as a long extension (Figure 7C). It is possible this inward-facing predicted region of the structure may interact with the tube, which can help with folding and assembly. 

### 3.6. Phage G Tail Sheath Helical Symmetry Compared to Other Known Phages

**Figure 8:**
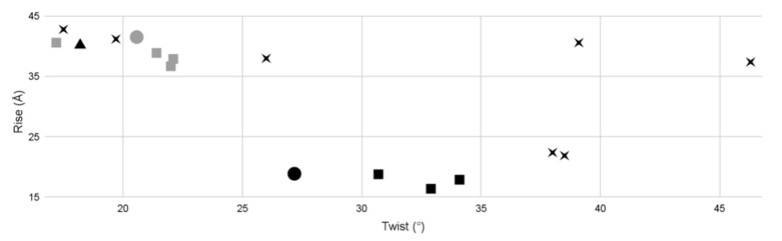
Helical symmetry parameters of the tails of various phages. The grey squares indicate the non-contracted tail sheath structures of myophages. The black squares indicate the contracted myophage structures of myophage. The black Xs indicate siphophages. The black triangle represents the T4 tail tube. The circles represent phage G non-contracted (grey) and contracted (black), as determined here. In this study, we included information from four myophages (T4, phi812K1-420, phiKZ, and phiRSL1) [6,10,11,33,43], and seven siphophages (lambda, YSD1, SPP1, Araucaria, T5, p2, and TP901-1) [35,36,37,38,39,40,41].

After obtaining the cryo-EM structure of the phage G non-contracted and contracted sheath, we then investigated how its helical symmetry parameters compared to existing structures of tailed phages. We collected available phage tail data from the literature and plotted the helical symmetry of all the data, including our results for the phage G tail sheath (Figure 8, Table 3).

In this comparison, we included information from four myophages (T4, Φ812, ΦKZ, and ΦRSL1) [12,13,35,36], and seven siphophages (λ, YSD1, SPP1, Araucaria, T5, p2, and TP901-1) [35,36,37,38,39,40,41]. The results show two clusters. The first cluster is for the non-contracted phage tail sheaths (grey squares and grey circle). The ranges in this cluster are 17–22° twist and 36–42.8 Å rise. All the non-contracted myophage tail sheath structures are found in this cluster. The siphophages λ, YSD1, and SPP1 are also shown in this cluster (Figure 8). In the second cluster, the contracted tail sheath structures are not as tightly grouped. The ranges for this cluster are 27.18–34.1° twist and 16.4–18.89 Å rise in Figure 8. In this cluster, only the myophage structures were grouped. The four phage tail structures falling outside of these two clusters were Araucaria, TP901-1, T5, and p2 (Figure 8).

The T4 tail is 925 Å long, and contracts to 420 Å [8,11,42]. The phi812 non-contracted tail is 2020 Å long, and contracts to 808 Å [11]. The phage G tail sheath is ~4500 Å long and contracts to 2040 Å. Although the length of phage G’s tail is more than two times longer than the phi812 tail and almost five times longer than the T4 tail, the tail sheaths of all three phages are six-stranded and right-handed helix tails, with relatively similar helical symmetry in the non-contracted and contracted states (Figure 8, Table 3); additionally, the contraction shortens the sheath length by a similar ratio. 

**Table 3:** Helical symmetry comparison among various phage tails.

Tail Morphology	Virus	Structure	Twist (°)	Rise (Å)	Reported Resolution (Å)	EMDB Entry	Host Gram (−/+)	Citation
Myophage	**phage G**	non-contracted sheath	20.57	41.53	7–8	25155	+	Current study
		contracted sheath	27.18	18.89	6–7	25154		
	**phi812K1-420**	non-contracted sheath	21.4	38.9	6.2	4051	–	[11]
		contracted sheath	30.7	18.8	4.2	4052		
	**phiRSL1**	non-contracted sheath	22.1	37.9	9.6	2244	–	[10]
	**phiKZ**	contracted poly sheath	34.1	17.9	19.0	5331	–	[33]
		non-contracted sheath	22	36.7	18.0	5332		
	**T4**	contracted sheath	32.9	16.4	N/A	N/A	–	[44,45]
		non-contracted	17.2	40.6	N/A	N/A		
		tube	18.2	40.2	3.4	8767		[45]
Siphophage	**SPP1**	tube	38.5	21.9	4.0	10792	+	[35]
	**YSD1**		19.7	41.2	3.5	22183	–	[38]
	**lambda**		17.5	42.8	6.4	20242	–	[46]
	**T5**		39.1	40.6	6.0	3692	–	[39]
	**p2**		46.3	37.4	22.0	2464	–	[42]
	**Araucaria**		26	38	24.0	2337	+	[36]
	**TP901-1**		38	22.4	20.0	2228	+	[47]

### 3.7. Evolutionary Analysis of Phage G Tail Sheath Protein gp178

We also performed a protein sequence-based evolutionary analysis of the phage G sheath protein gp178 [24]. To compare sheath proteins from an array of genetically diverged phages, the analyses were based on the more highly conserved *C*-terminal region of the sheath protein (residues 357–655 of T4 gp18). Evolutionary time was calibrated in time by congruence with a large terminase tree in the T4 and SPO1 clades. 

**Figure 9:**
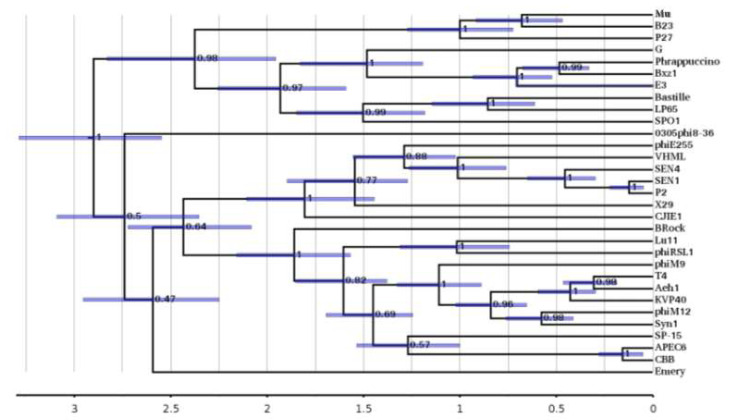
Phage G sheath timetree. A timetree bioinformatic analysis was conducted to analyze the evolutionary relationship among various phage tail sheath proteins (see Materials and Methods). The timeline unit used was 1.0 Gya. The error bars shown in purple report 95% confidence in the height of each node, and are accompanied by the posterior probability that all branches below the node are correctly placed.

These analyses found that—despite phage G’s sheath being structurally similar to that of T4 in both its helical symmetry (Figure 8) and subunit conformation (Figure 7)—the sheath proteins of the two phages are diverged in such a way that they likely separated in an ancestral split that occurred over 2.5 Gya (Figure 9). In addition, even the sheath proteins of what are currently the closest known phage G relatives (in phages Phrappuccino, Bxz1, and E3) are predicted to have evolved from an ancestral split that occurred almost 1.5 Gya. 

### 3.8. The Phage G Tail Sheath Gene Is Located in a Syntenous Tail Morphogenesis Gene Module

**Figure 10:**
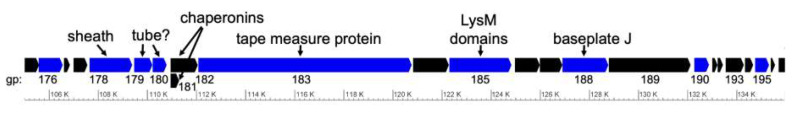
The phage G genome region containing the major tail morphogenesis genes. Gene products (gp), identified by mass spectrometry [4] in purified virions, are shaded blue.

The intriguing dichotomy of the phage G sheath’s similarities and differences to those of other myoviruses led us to seek a better understanding of other components of the phage G contractile tail. The phage G sheath gene is located toward the 5′ end of a module of genes (NCBI: NC_023719) whose arrangement is reminiscent of that observed in many (e.g., Mu [48], 0305phi8-36 [49], and ARV1 [50]), but not all, myoviral genomes. For example, downstream of the sheath gene is a pair of chaperonin genes, and immediately downstream of those is the tape measure protein (TMP) gene and a series of genes that likely encode baseplate components and tail fibers (Figure 10). The tail chaperonins (gp181 and gp182) were annotated as such due to the fact that they have a signature translational frameshift that was first described for the G-T genes of lambda [51]. Gp181 and gp182 were not detected in our previous mass spectral analyses of the proteins of phage G [4], consistent with their predicted function as chaperonins. 

However, other proteins encoded in the phage G tail morphogenesis region were identified by mass spectrometry. These proteins have other characteristics and/or sequence similarities (as determined by BlastP or HHpred) that strongly support their being components of the tail [4]. For instance, one of these proteins, gp183, has the classic characteristics of a TMP. These include its relatively great length (it is often the longest gene), which is a consequence of the TMP’s function as the tail length determinant (e.g., T4 [52] and TP901-1 [41]). To perform this role, a few copies (3–6) of TMP extend as an alpha helical structure from the baseplate to the neck region within the central core of a tail tube. Considering the function and length of the phage G tail (450 nm), it is not surprising that gp183 is 2893 residues long and has a predicted high overall content of α-helices (50%) and coils (44%) [4,53,54]. Phage G’s tail (4500 Å) and TMP length (2893 amino acids) are both approximately five times larger than T4’s tail (925 Å) [8,11,42] and T4’s TMP (gp29; 590 residues) [55]. 

The *C*-terminus of gp183 likely assists with genome ejection into the host cell by interacting with, and likely degrading, the cell wall—an additional function of the TMP in some phages [56]. This expectation is based on Blast and HHpred matches in the *C*-terminal region of gp183 with the LytD superfamily of beta-n-acetylglucosaminidases (residues 2439–2595, 1.99e-29) and Peptidase family M23 (residues 2622–2716, 1.20e-35). Other proteins encoded downstream of the TMP gene also had homology to domains and/or phage tail proteins that support their roles as baseplate and/or tail fiber proteins. For instance, gp188 has diverged similarity to the T4 baseplate wedge proteins gp6 (4.1e-28) and P2 gpJ (2.6e-25).

Currently, phage G has two tail tube candidates, gp179 (242 residues) and gp180 (189 residues), the genes for which are located between the sheath gene and the “G-T” chaperonins. This is the normal location for the tube gene in many myoviruses (Figure 10). Both gp179 and gp180 have diverged homology to a series of tube proteins, including those from diffocins (e.g., xkdM of the *Bacillus subtilis* prophage PBSX), myophages (e.g., gp19 of T4), and even siphophages (e.g., gpV of lambda), as determined by HHpred (all matches had probabilities > 95%). Whether phage G gp179 and gp180 both form part of the tube, or one of them forms the tube and the other has a different function (e.g., binding of the tube to the neck or baseplate), is unclear. 

## 4. Discussion

### 4.1. Unusual Tail Sheath Contraction in Phage G

Our EM of negatively stained phage G–host cells and our cryo-EM of purified phage G revealed that phage G’s tail contraction is different from that of typical myophage contraction. The former study revealed that all contracted tail sheaths at the tip of the tail were decoupled from the neck region (Figure 1). This is in contrast to the observations of T4 phage, the myophage “type phage”, in which the contracted tail sheaths were always coupled to the head–neck region after attachment to host cells. In this study, we assume that this difference is significant, even though phage G-like contraction can be induced in phage T4 by non-natural conditions [30]. 

The phage G sheath contraction toward the tail tip, as observed here, does not fit the current model based, on data from other myophages [7,11,57]. In the current model, irreversible tail sheath contraction is initiated by baseplate conformational change, itself initiated by tail fiber attachment to the host cell surface. This contraction propagates from the baseplate towards the head [6,9]. It is thought that this process transfers energy to the tail tube for puncturing the host cell’s membrane and cell wall to eject the dsDNA genome into host cell cytoplasm [7,11,32,57]. Assuming that the head–distal part of the tail initiates the signal to contract, the atypical contraction observed here suggests an alternative way of propagating this signal. One hypothesis is that propagation occurs along the tail-associated outer coils (Figure 4). These outer coils are not present in the other myophages discussed here. 

### 4.2. Missing Tail Sheath Anchor Point at the Neck Region in Phage G

**Figure 11:**
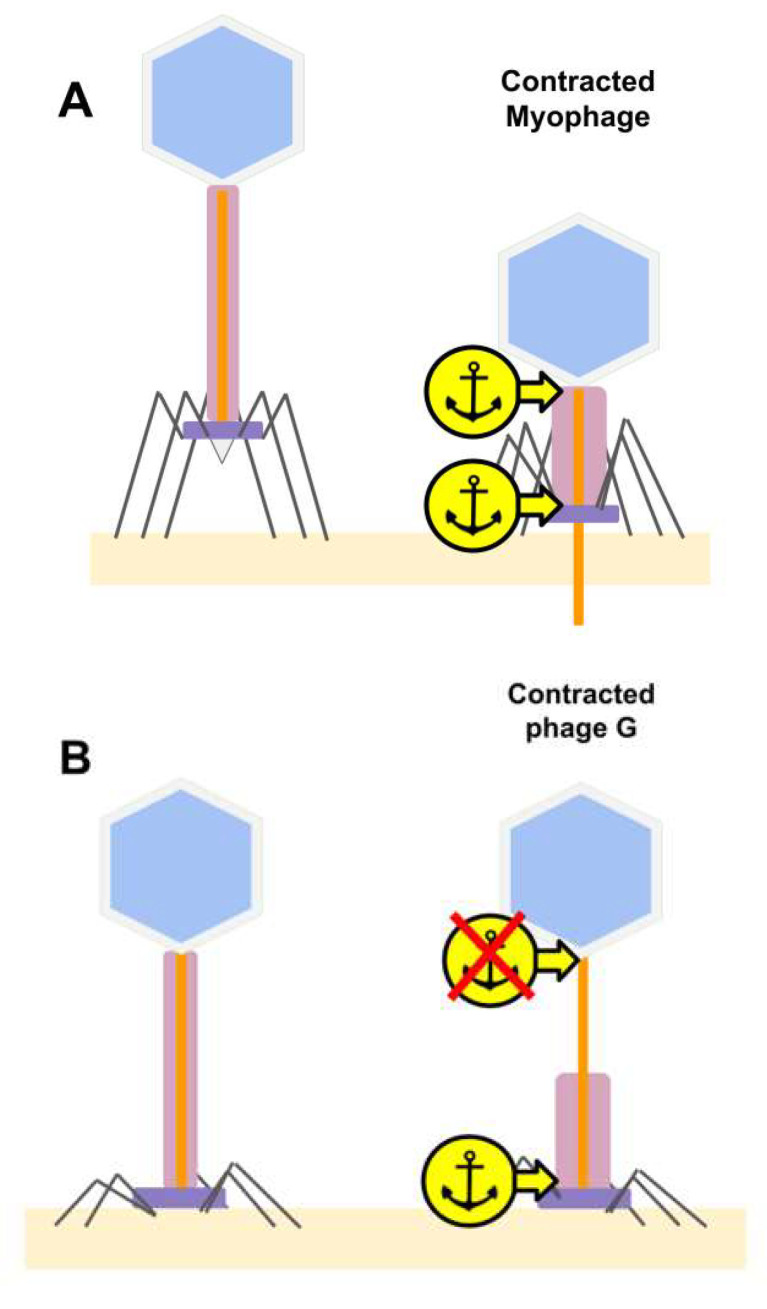
Tail sheath anchor points in a typical myophage and phage G during contraction. (**A**) In the typical myophage tail sheath contraction [5,58], the tail sheath stays in contact with the neck region via the upper of two initial anchor points. (**B**) In the atypical tail contraction of phage G, the upper anchor point appears to be missing and the contracted sheath is positioned at the tail tip (See Figure 1A).

As previously described [5,58], the typical tail sheath contraction for myophages is based on the tail sheath staying in contact with the head–neck region in all states. During the tail sheath contraction of a typical myophage, the tail sheath contracts upward toward the head, and this is proposed to help penetrate the inner tube through the host cell membrane and cell wall to proceed with infection [7,10,11], as shown in Figure 11A.

The location of a contracted tail can be controlled via anchoring proteins. Anchor points are described for T4 and illustrated in Figure 11 with the yellow arrows. In T4, anchoring proteins are described as follows: gp3, which contacts right before the tail terminator [53]; gp15, which helps with the head to tail attachment [54]; gp25, which is hypothesized to initiate contraction at the distal end, and has structural similarities to the sheath protein [59]. 

Our observations of the atypical tail contraction of phage G (Figure 1A) are illustrated in the cartoons in Figure 11, with the head-proximal anchor missing, while the distal anchor remains functional, holding the contracted tail sheath at the host cell surface. Loss of the upper anchor also causes a loss of capacity for protruding the tail tube mechanically. Furthermore, in the isolated phage G, as shown in our cryo-EM images (Figure 2), the contracted sheath would no longer be forced to stay at the tail tip due to the lack of attachment of the bottom end of the sheath/baseplate to the host cell surface but could instead freely slide and stay at an arbitrary location along the tail tube. We note that this anchor-point hypothesis was synthesized to explain our observations, but its structural and functional details remain to be established.

### 4.3. Evolutionary Implication of Phage Tail Mediated Infection Mechanisms and Future Directions

Our analysis of phage G’s tail morphogenesis gene region gives us more information about the structural components of its tail in relation to other myophages (Figure 10). Despite the similarities in the overall synteny of the phage G major tail gene module with those of other phages, and the functional assignment of a handful of genes, there remain more questions regarding the other phage G tail components. For instance: what is the full complement of genes required to form the tail, baseplate, and helical fibers? Based on the number of different proteins identified as part of the phage G virion, as well as precedents in other structurally complex myoviruses (e.g., SPO1, T4), it is likely that >20 different proteins are required to form the phage G tail. Similarly: what is the role of each of the phage G tail components? Research to address these questions is likely to generate novel findings as suggested by the existence of two, rather than one, phage G proteins (gp179, gp180) that have similarity to known tail tube proteins (Figure 10). 

There a are only a few reports, to our knowledge, that have mentioned a tail contraction behavior similar to phage G, where the contracted sheath can be found decoupled from the neck region. Negative stain images of isolated phages with contracted sheaths decoupled from the neck region were found for *Listeria monocytogenes* phage 0176 [57], and three phages infecting *Burkholderia pseudomallei*, KS5, KS14 [60], and ST2 [61], as well as T4 in non-natural conditions [29,30]. In this report, we give the first structural insight into the contraction mechanism and protein structure of the sheath in phage G to probe the contraction states we have observed. We find similarities and evolutionary relationship to other myophages as follows: (1) The organization of genes in tail morphogenesis region of the genome; (2) Sequences of genes for the sheath proteins; (3) Cryo-EM structures of the tail sheath at multiple levels— the helical arrangement of the entire sheath and the protein fold of the sheath protein; (4) Structural changes upon contraction. Thus, whatever addition must be made to the current model for myophages, this addition is not likely to be correlated with these four aspects of tail contraction [7,10,11]. However, many follow-up studies will be needed to clarify how phage G uses its tail to attach to the host cell surface and inject its DNA genome into host cell cytoplasm, and if the atypical tail contraction observed in this study is functionally relevant in infecting host cells. 

## Figures and Tables

**Table 1:** Tail contraction and head states from cryo-EM micrographs.

Capsid State	Non-Contracted	Contracted Tail Location		
		Near Head	Middle	Near Tip
DNA full	196	25	3	104
Partial DNA	3	1	0	7
Empty	12	1	0	12

**Table 2:** Cryo-EM helical structure details of phage G tail sheath in non-contracted versus contracted states.

Helical Structure Features	Non-Contracted	Contracted
Outer diameter (Å)	240	320
Inner diameter (Å)	60	120
Pitch (Å)	706.01	245.57
Rise (Å)	41.53	18.89
Twist (°)	20.57	27.13

## Data Availability

The 3D maps of the non-contracted and contracted tail sheaths are deposited to the Electron Microscopy Data Bank (EMDB) with accession IDs, EMD-25155 and EMD-25154, respectively.

## Supplementary Materials

The following are available online at https://www.mdpi.com/article/10.3390/v13102094/s1, Figure S1: Phage G tail sheath reconstruction FSC curves, Figure S2: The contracted phi812 sheath subunit density compared to the contracted phage G sheath subunit density, Table S1: Data collection parameters of phage G cryo-EM dataset, Table S2: Image processing details of the phage G helical tail sheath reconstruction.
