# Supplementary material for: Structural Studies of the Phage G Tail Demonstrate an Atypical Tail Contraction
Source: Viruses. 2021 Oct 18;13(10):2094. doi: 10.3390/v13102094 (PMC8570332; doi:10.3390/v13102094)
Supplement: Supplementary file 1 [file viruses-13-02094-s001.zip › 20210728_Article_Viruses_Gonzalez_et_al_Supplement.pdf]

Supplemental Figures

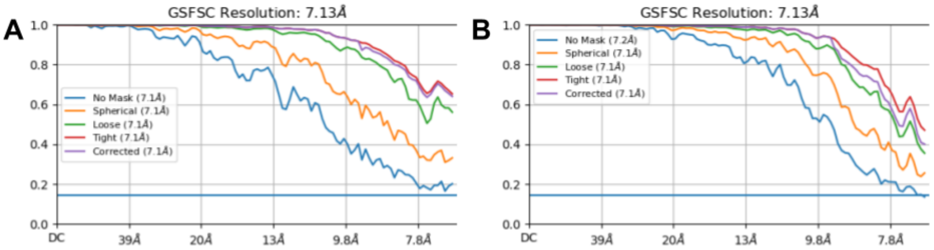

Supplementary Figure S1. Phage G tail sheath reconstruction FSC curves. The non-contracted and contracted structures were obtained using the details described in the methods section and Supplementary Table 2. The FSC plots shown here were generated from CryoSPARC [1] for the non-contracted (A) and contracted (B) phage G tail sheath structures. The FSC curves remain significantly above zero at the Nyquist (e.g. the right-edge of plots) due to the resolution of the 3D reconstructions reaching the Nyquist resolution of the images (6.968 Å for the physical pixel size of 3.484 Å). Reconstructions using the original super-resolution images were not possible due to the loss of original movies after motion correction that saved 2x binned movie averages.

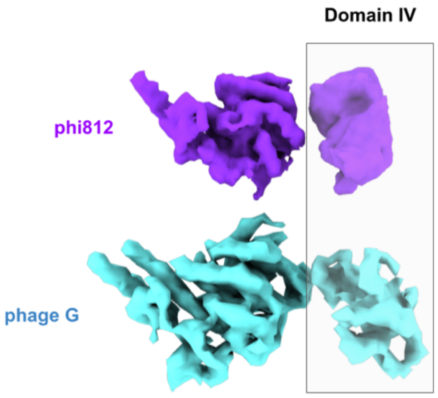

Supplemental Figure S2. The contracted phi812 sheath subunit density [2] compared to the contracted phage G sheath subunit density. Using Chimera's Segger [3], the sheath subunit density for both structures was isolated and aligned for comparison. The resolution of the outer domain IV in both structures is less well resolved as shown within the grey box region.

Supplementary Table S1. Data collection parameters of phage G cryo-EM dataset

Parameters	Data collection
Nominal magnification	8700
Voltage (KeV)	300
Electron exposure (e −/Å2)	14.5
Defocus range (μm)	1–5
Detector	K2
Movies	375
Beam size (μm)	3.35

Supplementary Table S2. Image processing details of the phage G helical tail sheath reconstruction

Image Processing	Non-contracted	Contracted
Software for reconstruction	Relion [4]; CryoSPARC [1]	
Pixel Size (Å/px)	3.484	
Box Size (px)	224	
Interbox distance (Å)	93.5	
Starting particles	22,755	
Rounds of 2D classification	6	
Final particles after helical refinement	2,099	5,358
Symmetry applied	C6	C6
Refined twist (º)	20.570	27.130
Refined rise (Å)	41.530	18.890

References

1. Punjani, A., et al., *cryoSPARC: algorithms for rapid unsupervised cryo-EM structure determination.* Nat Methods, 2017. **14**(3): p. 290-296.
2. Nováček, J., et al., *Structure and genome release of Twort-like Myoviridae phage with a double-layered baseplate.* Proceedings of the National Academy of Sciences, 2016. **113**(33): p. 9351-9356.
3. Pintilie, G. and W. Chiu, *Comparison of Segger and other methods for segmentation and rigid-body docking of molecular components in cryo-EM density maps.* Biopolymers, 2012. **97**(9): p. 742-60.
4. He, S. and S.H.W. Scheres, *Helical reconstruction in RELION.* J Struct Biol, 2017. **198**(3): p. 163-176.
